# Structure-based discovery of potentially active semiochemicals for *Cydia pomonella* (L.)

**DOI:** 10.1038/srep34600

**Published:** 2016-10-06

**Authors:** Jiyuan Liu, Zhen Tian, Yalin Zhang

**Affiliations:** 1 Key Laboratory of Plant Protection Resources & Pest Management of the Ministry of Education, College of Plant Protection, Northwest A&F University, Yangling 712100, Shaanxi, China; 2 Department of Medicinal Chemistry, School of Pharmacy, Fourth Military Medical University, Xi’an 710032, Shaanxi, China

## Abstract

The development of physiologically active semiochemicals is largely limited by the labor-consuming searching process. How to screen active semiochemicals efficiently is of significance to the extension of behavior regulation in pest control. Here pharmacophore modeling and shape-based virtual screening were combined to predict candidate ligands for *Cydia pomonella* pheromone binding protein 1 (CpomPBP1). Out of the predicted compounds, ETrME displayed the highest affinity to CpomPBP1. Further studies on the interaction between CpomPBP1 and ETrME, not only depicted the binding mode, but also revealed residues providing negative and positive contributions to the ETrME binding. Moreover, key residues involved in interacting with ETrME of CpomPBP1 were determined as well. These findings were significant to providing insights for the future searching and optimization of active semiochemicals.

Chemical communication in insects takes place by perceiving a myriad of semiochemicals in the olfaction system^1^,^2^,^3^,^4^. Actually, chemical sensing is involved in dominant physiological behaviors of insects, including mating, feeding, laying eggs as well as avoiding threats^4^,^5^,^6^. Semiochemicals active to insects are suggested to be detected and translated into nerve impulses in antennal sensilla^1^,^7^,^8^. Odorant binding proteins (OBPs), highly concentrated in antennal sensilla lymph, are regarded to be the first proteins to interact with pheromones before ferrying these molecules to corresponding odorant receptors (ORs) expressed on the membrane of olfactory sensory neurons^9^. Other elements like sensory neuron membrane proteins (SNMPs) and odorant-degrading enzymes (ODEs) were also required for odorant-evoked response and might contribute to the rapid activation and termination of odorant-mediated behaviors^8^,^10^,^11^,^12^.

Leal in 2005 advanced the notion of “reverse chemical ecology”, a new concept for semiochemicals discovery based on the binding ability of olfaction related proteins rather than the bioassays of insect behaviors^11^. Prior studies revealed that the specificity of insect olfaction system heavily depends on ORs, therefore ORs are considered as the best target for semiochemicals searching^10^,^13^. For the development of novel drugs, drug-receptor interactions are typically applied to construct protein-based approaches in pharmaceutical industries^14^,^15^. However, what should be confessed is that searching for potentially active semiochemicals based on *in vitro* binding studies with ORs is technically infeasible, especially when considering that functional expression of ORs is far from easy^11^,^16^. Due to the special role of OBPs in insect olfaction system, small molecules which cannot bind to OBPs are incapable of reaching the membrane, let alone evoking certain behaviors of insects. So the best studied OBPs are advisable to be chosen as alternative targets for the discovery of semiochemicals^17^,^18^,^19^.

Recently, more and more researches begin to realize the significances of incorporating virtual screening into semiochemicals searching^18^,^19^. Nevertheless, this study pioneers the application of pharmacophore modeling in searching physiologically active semiochemicals at the best of our knowledge. Along with its advances, pharmacophore model has evolved to represent the spatial arrangements necessary for a small molecule to interact with the target protein^20^. By incorporating the geometric and chemical features of known active ligands (including inhibitors or activators), pharmacophore modeling has been widely used in the high performance screening and rational design of novel lead compounds^21^,^22^,^23^. Moreover, pharmacophore modeling and other methods like shape-based virtual screening are usually combined to increase the accuracy of prediction^21^,^22^.

As a quarantine pest, *Cydia pomonell*a causes severe damage to fruit production throughout the world every year. The great advantages of disrupting insect behaviors make it a promising way in controlling fruit pests. However, for *Cydia pomonella*, the widely used attractant, Codlemone (sex pheromone of *Cydia pomonella*), is only active to male moths, searching for broad-spectrum attractants or repellents is necessary to control *Cydia pomonella* more efficiently and nuisanceless. The aim of this study is to discover potentially active semiochemicals for *Cydia pomonella* through pharmacophore-based virtual screening. As a subfamily of OBPs, pheromone binding proteins (PBPs) are indispensable in regulating insect behaviors related to reproduction. According to our previous researches^19^,^24^, CpomPBP1 (pheromone binding protein 1 from *Cydia pomonella*) may act as the transporter of Codlemone, the major sex pheromone of *Cydia pomonella* which presents high affinity to CpomPBP1 but poor affinity to other *Cydia pomonella* PBPs like CpomPBP2. With respect to this, it is advisable to develop pharmacophore model using structural data on the key interactions between CpomPBP1 and Codlemone. In the present study, pharmacophore-based virtual screening is used in combination with other three methods including Gaussians molecule shape similarity, binding affinity calculation and *in vitro* binding assay to discover potentially active semiochemicals for *Cydia pomonella*. Thereafter, to study the binding mode between CpomPBP1 and the molecule owning the highest affinity, molecular simulations (molecular dynamics, per residue free energy decomposition and alanine scanning mutagenesis) and experimental methods (site-directed mutagenesis and *in vitro* binding assay) are used jointly. Pharmacophore-based virtual screening provides a convenient way to search large chemical database and increases the chance of target hitting. What’s more, the revelation of binding mode (driving forces of binding, key residues, etc.) is of guiding importance to the rational design of novel active semiochemicals.

## Results and Discussion

### Structure-based pharmacophore modeling

**Figure 1:**
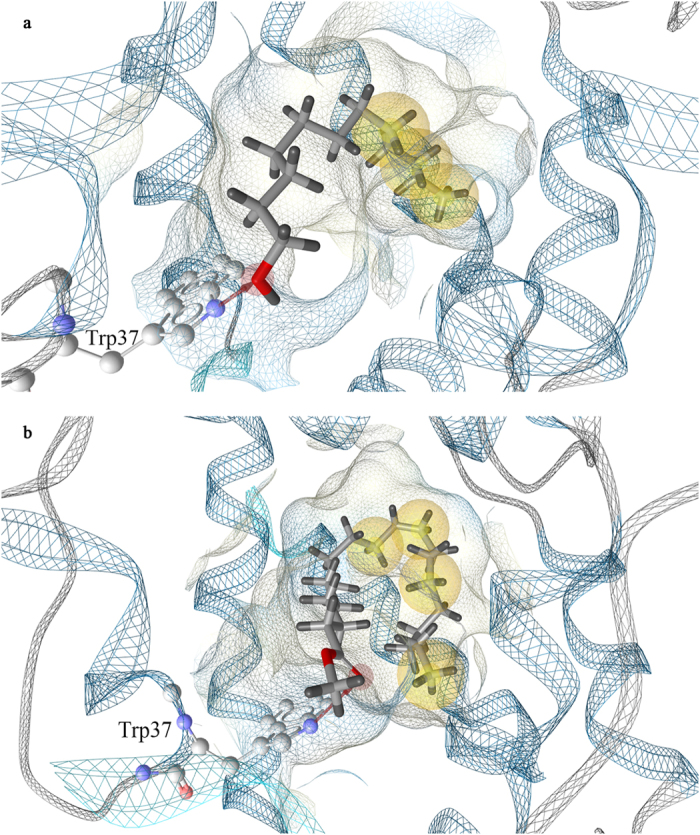
Structure-based pharmacophore modeling. (**a**) Pharmacophore model based on the binding mode of CpomPBP1-Codlemone complex. The model consists of three hydrophobic features (yellow) as well as one hydrogen bond (red arrow). (**b**) Pharmacophore model based on the binding mode of CpomPBP1-ETrME complex. The model consists of four hydrophobic features (yellow) as well as one hydrogen bond (red arrow).

In the current study, CpomPBP1-Codlemone complex was constructed based on the homology 3D model of CpomPBP1 (Figure S1a), key interactions between Codlemone and CpomPBP1 derived from the constructed complex were then transformed into a hypothesis of pharmacophore by LigandScout4.09. As shown in Fig. 1a, the pharmacophore model was characterized by 4 features: one hydrogen bond acceptor (HBA) and three hydrophobic groups. Several excluded volumes localized in the space regions where the backbones or sidechains of residues lie were automatically generated in the model, reflecting that these regions were inaccessible to any potential ligand due to possible steric restrictions. The HBA feature represented carbonyl group of Codlemone which accounted for the hydrogen bond interaction with NH atom of residue Trp37 sidechain. The three hydrophobic groups were occupied by olefin groups and aliphatic chain of Codlemone.

In order to verify the pharmacophore features as hot spots for the interaction between CpomPBP1 and Codlemone, Molecular Interaction Fields (MIFs) produced by EASYMIFs was employed to identify CpomPBP1 structure regions showing high propensity for the interaction with ligands^25^,^26^. Chemical groups CMET (carbon of CH3-group, hydrophobic) and OA (hydroxyl) were selected as probes to explore hydrophobic interactions and to mimic hydrogen bond acceptor, respectively. According to the energy map derived from the interaction between CpomPBP1 and these two probes (Figure S2), the golden and slate blue points indicated areas providing the most favorable interaction energies. The points of MIFs agreed well with above pharmacophore features, suggesting credibility of the pharmacophore model prepared for virtual screening.

### Prospective Virtual Screening

Based on the pharmacophore model derived from CpomPBP1-Codlemone complex, pharmacophore-based virtual screening of the candidate semiochemical screening library (~3233 compounds) were carried out employing LigandScout Fast Flexible Search algorithm. According to the standard that only those matching all the pharmacophore features were considered as hits, 180 ones were obtained, of which some hits were repeatedly contained due to their different protonation states. For the repeated ones, only those possessing the highest pharmacophore-Fit score were kept. Finally, as shown in Table S1, 133 compounds were put into further analysis.

To improve the accuracy of prediction, we also performed molecule shape alignment applying the Gaussian Shape module of LigandScout. With the Codlemone conformation extracted from CpomPBP1-Codlemone complex being query, the 133 hits were subsequently ranked by Gaussian Shape Similarity Score based on shape overlap and chemical features. The obtained 31 compounds (Table S2) were then submitted to LigandScout to calculate their binding affinities towards the binding sites of CpomPBP1. Theoretically, higher Binding Affinity Score is often coupled with higher binding affinity to protein. Considering the high costs of compounds listed in Table S2, we set the threshold of activity cutoff to 30.00 kcal/mol (Binding Affinity Score >30.00 kcal/mol) to reduce the total number of compounds for further *in vitro* binding assays. Consequently, 18 unique compounds (Table S3) from the candidate semiochemical screening library were finally predicted to be candidate compounds in the prospective virtual screening.

### Competitive binding assay and dissociation constants calculation

**Figure 2:**
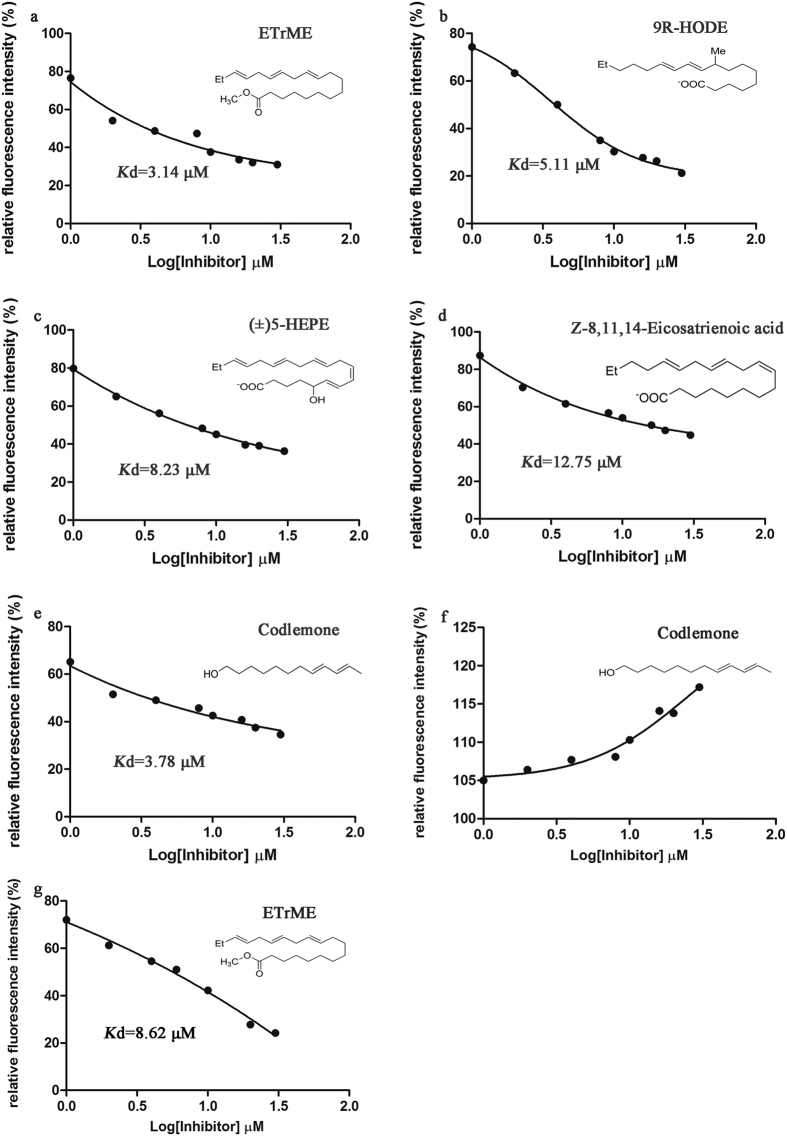
Binding curves of tested ligands to CpomPBP1 and CpomPBP2. (**a**–**e**) Binding curves of ETrME, 9R-HODE, (±)5-HEPE, Z-8,11,14-Eicosatrienoic acid and Codlemone to CpomPBP1. These 5 ligands showed relatively high affinity to CpomPBP1 with corresponding dissociation constants (*K*_d_) being 3.14 μM, 5.11 μM, 8.23 μM, 12.75 μM and 3.78 μM, respectively. (**f**,**g**) Binding curves of Codlemone and ETrME to CpomPBP2. In the titration course, Codlemone exhibited no binding, whereas ETrME could bind to CpomPBP2 with *K*_d_ being 8.62 μM.

Competitive binding assay was performed to test the accuracy of prediction and to measure the affinity of each candidate compound towards CpomPBP1. Our pre-test revealed ethanol of GC grade was advisable to be used as solvent in the competitive binding assay if only its volume fraction was not beyond 10% (Figure S3). As shown in Fig. 2 and Figure S4, 4 out of the candidate ligands including ETrME (ZINC31983243), 9R-HODE (ZINC27643501), (±)5-HEPE (ZINC12496534) and Z-8,11,14-Eicosatrienoic acid (ZINC13507101) were detected to bind to CpomPBP1 with their dissociation constants (*K*_d_) being 3.14 μM, 5.11 μM, 8.23 μM and 12.75 μM, respectively. Particularly, with respect to the affinity to CpomPBP1, ETrME behaved the best, even better than Codlemone (Fig. 2a,e). Unlike Codlemone, the selection of ETrME between CpomPBP1 and CpomPBP2 was not that significant. Our binding results revealed that ETrME also exhibited high affinity to CpomPBP2 (Fig. 2g), even though the *K*_d_ of CpomPBP2-ETrME system (*K*_d_ = 8.62 μM) was about 2 times higher than the counterpart of CpomPBP1-ETrME system (*K*_d_ = 3.14 μM). Such a difference suggested that different mechanisms were involved in the interactions between CpomPBP1 and Codlemone/ETrME, although ETrME was discovered based on the CpomPBP1-Codlemone interaction.

In the current study, to discover active compounds that can be potentially used in modulating behaviors of *Cydia pomonella*, it is important to identify whether these 4 active compounds possess the typical characteristic of odorant. Traditionally, to be an odorant, the small molecule should be easy to volatize. For the 4 compounds plus Codlemone (as a positive control), an estimation of main physical constants characterizing the substance transition from liquid into gaseous state (boiling point, vapor pressure and enthalpy of vaporization) was performed by ACD/I-Lab version 2.0^27^. Intriguingly, the enthalpy of vaporization of ETrME is only 1.00 kcal/mol more than that of Codlemone (Table S4). Additionally, like Codlemone, ETrME are liquid as well at room temperature (Table S4). All these indicate that ETrME is potentially to be an odorant and could be treated as a lead for rational semiochemical design.

### Stability analysis of the CpomPBP1-ETrME complex

**Figure 3:**
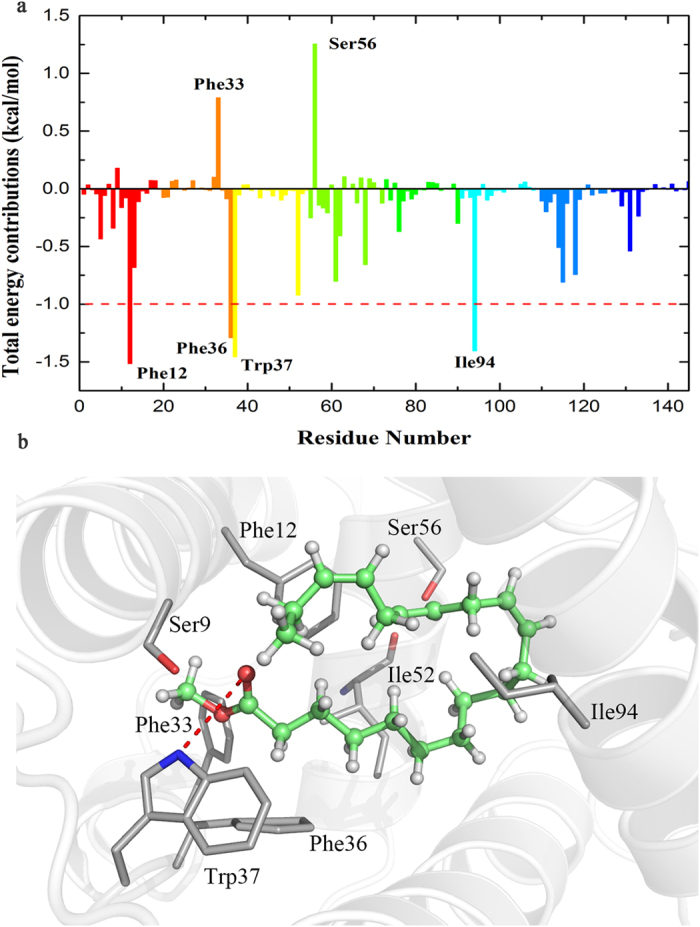
The interaction between CpomPBP1 and ETrME. (**a**) Residue-ligand interaction spectrum of CpomPBP1- ETrME complex according to the MM-PBSA method. The x-axis denotes the residue number of CpomPBP1 and the y-axis denotes total interaction free energy contribution of each residue. (**b**) Key interactions and H bond patterns at the active site observed during MD simulations of ETrME. ETrME was presented with the stick-and-sphere model. Color code: green, C; red, O; white, H. Key residues are presented with stick model. Color code: gray, C; red, O; blue, N; white, H; red dashed line, H bond.

*In vitro* binding assay showed that CpomPBP1 exhibited the highest affinity to ETrME (the lowest *K*_d_), so CpomPBP1-ETrME complex was subjected to molecular dynamic (MD) simulations for which the whole process lasted 75 ns. The complex stability was assessed by root-mean-square deviation (RMSD) of the backbone atoms in CpomPBP1-ETrME complex. As shown in Figure S5a, the complex achieved equilibrium at ~12.5 ns with average RMSD value being 3.51 Å, and the conformation of ETrME in the complex fluctuated very small with RMSD value around 3.98 Å along the process of MD simulations (Figure S5b). Moreover, root-mean-square fluctuation (RMSF) was used to depict the flexibility and local motion characteristics of CpomPBP1-ETrME complex. In Figure S5c, most residues composing the binding pocket of CpomPBP1 (Fig. 3b) exhibited little RMSF fluctuation. Based on the average-linkage algorithm and the pairwise RMS (root mean square), clustering analysis of the 75 ns MD simulation trajectory produced 5 clusters in CpomPBP1-ETrME complex (Table S5). The MD representative structure Cluster 5 (Figure S6) which had the highest occurrence resembled its conformation in the docking structure, their structures superimposition revealed the smallest conformational variation with RMSD being 1.13 Å (Table S5), suggesting that the whole MD simulations reflected correct motion behavior of CpomPBP1-ETrME complex. All of these indicated stability of the CpomPBP1-ETrME complex in the course of 75 ns MD simulations.

### Free energy decomposition for CpomPBP1-ETrME complex

To reveal residues contributing remarkable total interaction free energy to the formation of CpomPBP1-ETrME complex, the per residue free energy contribution spectrum was illustrated in Fig. 3a. It can be seen that 4 residues including Phe12, Phe36, Trp37 and Ile94 contributed more than 1.00 kcal/mol to the total interaction free energies. According to Fig. 3a and Table S6, the sidechains of residues (Phe12, Phe33, Phe36, Trp37, Ile 52 and Ile94) composing the hydrophobic pocket of CpomPBP1 contributed prominent van der waals (VDW) energy to the total interaction energies, residues Phe12 and Phe36 in particular provided more than 2.00 kcal/mol. Residue Trp37 also made a significant favorable electrostatic energy contribution with a high value of −1.61 kcal/mol. It is not surprising since residue Trp37 could form a hydrogen bond (H-bond) interaction with ETrME (Fig. 3b) with an average distance of 3.10 Å between NE1 atom (HH11) of Trp37 sidechain and carbonyl oxygen atom (O21) of ETrME along the whole 75 ns MD simulations (Figure S7). As shown in Table S7, the H-bond occupancy rate reached 74.84% for the two atoms (NE1 and O21). Dynamic stability of crucial VDW interactions described above were also monitored along the simulation time by measuring the atomic distances between C17 atom of ETrME and CZ atoms derived from the residues Phe12 and Phe36 (Figure S7). As shown in Figure S7b, the VDW interaction between ETrME and the sidechain of Phe36 is dynamically stable and strong (red spectrum, average distance = 3.8 Å), while the one existed between Phe12 sidechain and ETrME is also dynamically stable but a little weak due to the relatively long atom distance (black, average distance = 4.5 Å). By analyzing dynamic interactions of CpomPBP1-ETrME complex, it can be concluded that all key interactions were stable, implying that the complex occurred no obvious conformational transformation throughout the whole MD simulation process. Due to the remarkable polar solvation energies (negative to ligand binding), the total interaction energies of Ser9, Phe33 and Ser56 were unfavorable to the binding of ETrME to CpomPBP1, even though strong VDW interactions were detected between these three residues and ETrME (Table S6, Figure S8). Based on the analysis for the MD representative structures of CpomPBP1-ETrME complex, we found that the O atom derived from the mainchain of residue Ile52 dynamically stabilized the orientation of hydroxyl group from the sidechain of residue Ser56. While the orientation of hydroxyl group from the Ser9 sidechain was dynamically stabilized by the NE1 atom derived from the sidechain of residue Trp37. The orientation of Ser9 may cause a tiny proportion of fluctuation in the H-bond interaction between Trp37 and ETrME. Furthermore, as observed from the MD representative structure of CpomPBP1-ETrME complex, the conformation of Phe33 sidechain could produce steric impact on the methoxy group of ETrME. For further design of semiochemicals, the unfavorable interactions described above should be avoided.

### Alanine mutations reveal crucial residues

The favorable interaction residues contributing more than 1 kcal/mol (Phe12, Phe36, Trp37, Ile52 and Ile94) and the unfavorable interaction residues (Ser9, Phe33 and Ser56) were subjected to computational alanine scanning mutagenesis (ASM) method (Figure S8). As shown in Table 1, the mutation of Phe12 (F12A), Phe36 (F36A) and Trp37 (W37A) caused remarkable change in the binding free energy (ΔΔG_bind-cal_) of CpomPBP1-ETrME complex, with changed values being 4.13 kcal/mol, 4.55 kcal/mol and 5.21 kcal/mol, respectively. However, two relatively lower values of ΔΔG_bind-cal_ were produced by changing Ile52 (I52A, 2.31 kcal/mol) and Ile94 (I94A, 3.23 kcal/mol) each into alanine. The ASM results also clearly indicated that the mutation of Ser9 and Ser56 shed little impact on the ETrME binding with corresponding binding free energy changes less than 1.00 kcal/mol (Table 1). Interestingly, we found that CpomPBP1-ETrME interactions were enhanced when mutating Phe33 into Ala with the total interaction free energy being changed by more than 2.00 kcal/mol. Based on the theoretical free energy changes caused by alanine mutations, residues Phe12, Phe36 and Trp37 were successfully predicted as hot-spots in the CpomPBP1-ETrME complex.

**Figure 4:**
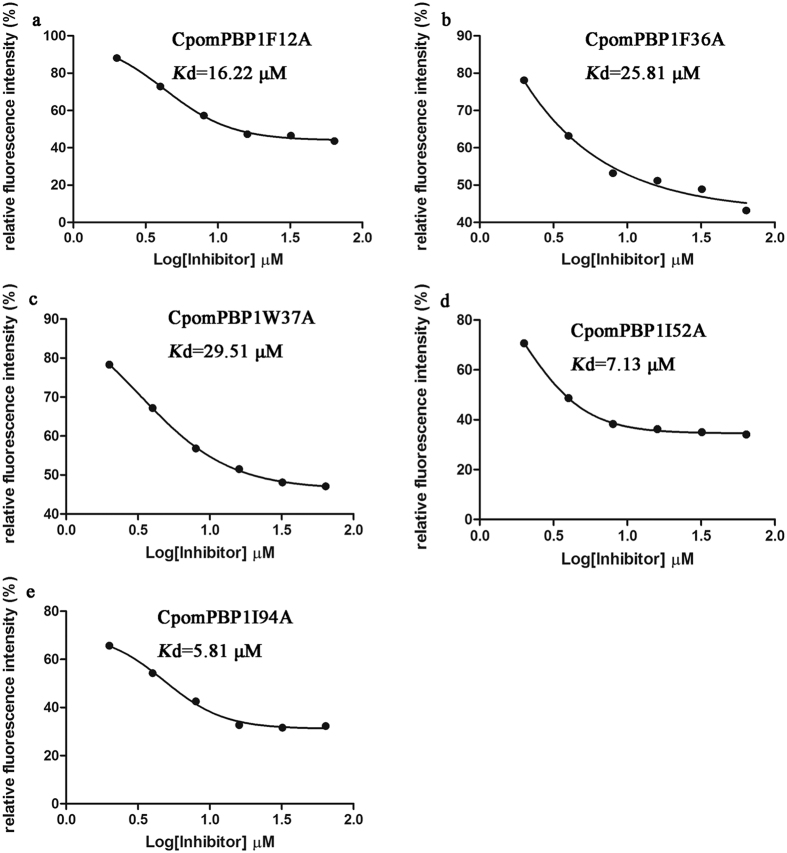
Binding curves of ETrME to mutant types of CpomPBP1. The mutation of each site decreased the binding ability of CpomPBP1 to varying degree.

To check the results of ASM, the 5 mutant types of CpomPBP1 protein (CpomPBP1F12A, CpomPBP1F36A, CpomPBP1W37A CpomPBP1I52A, and CpomPBP1I94A) were obtained based on site-directed mutagenesis (Figure S9) and subjected to competitive binding assay. As for the three residues (Ser9, Phe33 and Ser56) negative to the binding of ETrME, they were not put into site-directed mutagenesis due to their relatively lower ΔΔG_bind-cal_. As shown (Fig. 4), the binding ability of mutant CpomPBP1 exhibited decrease of varying degree. Thereafter, the experimental binding free energy changes (ΔΔG_bind-exp_) of each mutant CpomPBP1 were calculated and listed in Table 1. It was evident that the deviation between ΔΔG_bind-cal_ and ΔΔG_bind-exp_ was inside the acceptable range. More importantly, the results also showed that Phe36 and Trp37 met the criterion of hot-spot. This was partially in agreement with the ASM results with Phe12 being an exception. According to our binding assay, residue Phe12 fell into the range of warm-spot. However, the difference between ΔΔG_bind-cal_ and ΔΔG_bind-exp_ of CpomPBP1F12A was not beyond the acceptable limits.

In conclusion, considering the results of ASM and competitive binding assay in combination, it was apparent that Phe36 and Trp37 were two key residues associated with the interaction between CpomPBP1 and ETrME.

## Conclusions

This study for the first time introduced pharmacophore modeling to semiochemicals discovery, and successfully discovered a compound (ETrME) behaved better than Codlemone in the aspect of binding to CpomPBP1. Following studies on the interaction between CpomPBP1 and ETrME conducted by molecular docking, per residue free energy decomposition, ASM and site-directed mutagenesis provided a valuable perspective on the rational design of novel semiochemicals. Here present some important finds: hydrophobic interactions derived from the sidechains of Phe12, Phe36, Trp37, Ile52 and Ile94, especially the former two residues, played a vital role in enhancing the binding affinity of ETrME. The H-bond formed between Trp37 and ETrME was crucial to reinforce the ligand binding. The results of ASM and site-directed mutagenesis jointly revealed residues Phe36 and Trp37 were hot-spots to be exploited to improve the binding affinity of ETrME-based semiochemicals. Finally, it was notable to mention that residues Ser9, Phe33 and Ser56 around CpomPBP1 binding sites provided negative forces to the binding of ETrME. For novel semiochemical design, the interactions between these three residues and semiochemicals should be avoided in case of reducing binding affinity.

Within this study, features of the structure-based pharmacophore model derived from MD trajectories of CpomPBP1-ETrME complex were also depicted using LigandScout (Fig. 1b). As shown in Fig. 1, both pharmacophore models were characterized by the hydrogen bond formed with residue Trp37. However, slightly different interaction patterns that could occur in the binding pocket of CpomPBP1 were present. Due to longer aliphatic chain group of ETrME, the pharmacophore model derived from CpomPBP1-ETrME complex represented an additional hydrophobic feature at bottom of the hydrophobic pocket (Fig. 1b). It was evident, at least for CpomPBP1, that ligand hydrophobicity shed larger impacts on ligand binding in comparison with its aliphatic chain length.

The dependency of insect behaviors on semiochemicals provides possibility to control pests by disrupting their behaviors. However, active semiochemicals discovery is traditionally labor- and time-consuming, which largely lagged the development and application of insect behavior modulators. To solve this problem, pharmacophore-based virtual screening method was introduced in the present study. Our results suggested that current pharmacophore models derived from the two CpomPBP1-participated complexes were useful templates for semiochemicals discovery. Comparing with the widely used Codlemone in controlling and monitoring *Cydia pomonella*, the newly discovered ETrME possessed advantages of higher affinity to CpomPBP1 and lower cost, which made it a promising lead in designing and modifying more effective semiochemicals for *Cydia pomonella*. The pharmacophore-based virtual screening was validated to be an effective method in discovering active semiochemicals, it could not only largely simplify the process involved in searching for active semiochemicals, but also increase the chance of semiochemical discovery by searching larger chemical database.

## Methods

### Construction of the candidate semiochemicals screening library

As a major sex pheromone component of *Cydia pomonella*, Codlemone specifically exhibited high affinity to CpomPBP1, indicating that CpomPBP1 was the possible transporter of Codlemone^19^,^24^. So to construct the candidate semiochemical library for CpomPBP1 mediated virtual screening, the 2D structure of Codlemone was advisable to be a probe for compounds searching in the ZINC database version 12 (~30,000,000 compounds)^28^. By reference to the Structure Similarity Search in ZINC, chemicals with more than 70% similarity to the probe were output in SDF format. The output compounds were generated in 4 pH ranges including medium pH (6.0 to 8.0), high pH (8.0 to 9.5), low pH (4.5 to 6.0) and pH = 7.0 form. The collection of obtained compounds composed the candidate semiochemicals screening library.

### Structure-based pharmacophore modeling and virtual screening

Homology modeling and molecular docking were employed to construct the 3D structure of CpomPBP1 and CpomPBP1-Codlemone complex^29^,^30^. All details of the construction procedure were performed according to our previous reports^19^,^31^. The structure-based pharmacophore model derived from CpomPBP1-Codlemone complex was then constructed with LigandScout 4.09^32^ and used for the screening of candidate semiochemical screening library.

All small molecules in the library were converted into a collection of 3D structures using the Icon tool provided by LigandScout 4.09, a maximum number of 200 conformations for each molecule were generated applying the Icon best option so as to reproduce the flexibility of molecules during the virtual screening^33^,^34^. The energy window parameter and the RMS threshold were set to 20 kcal/mol and 0.8, respectively^35^. The candidate semiochemical screening library was stored in the LigandScout database format (LDB). The pharmacophore-based virtual screening was performed using the Iscreen module provided by LigandScout^26^, and pharmacophore-Fit score was employed to rank the compounds. It is the molecule matching all query features that was considered as a hit (Max number of omitted features was set to 0). The exclusion volume spheres were also checked to take into account the spatial constraints of CpomPBP1 protein binding site in the virtual screening step. To increase the accuracy of prediction, shape-based overlay of chemical structures was utilized as well. In this part, molecules with Gaussian Shape Similarity Score less than 0.50 were wiped off. After the filtration of former two steps, remaining molecules were subjected to binding affinity calculations, only those exhibiting Binding Affinity Score beyond 30 kcal/mol were kept.

### Competitive binding assay

According to the results of virtual screening and commercial availability of the matched semiochemicals, 8 compounds (including Codlemone) were finally subjected to competitive binding assay by taking 1-NPN as the fluorescence probe. All ligands including 1-NPN were dissolved in ethanol of GC grade. To measure the binding affinity between compounds and CpomPBP1, 50 mM Tris-HCl (pH 7.0) containing 2 μM CpomPBP1 and 2 μM 1-NPN was titrated with each compound (final concentration ranging from 0 to 64 μM). The fluorescence was measured on a Hitachi F-4600 spectrofluorimeter with a slit width of 5 nm for excitation and emission. The excitation wavelength was decided to be 337 nm and the fluorescence emissions were recorded from 350 to 500 nm. The *in vitro* binding assays between CpomPBP2 and small molecule compounds were performed in the same way.

### Molecular dynamic simulations and computational alanine scanning

The complex of CpomPBP1-ETrME was constructed by molecular docking simulations using the program GOLD5.3^30^. All molecular dynamics (MD) simulations for CpomPBP1-ETrME complex were performed with Amber12 package^36^. The parameters and charges of ETrME were optimized by the GAFF and the AMI-BCC method^37^,^38^. The AMBER for bioorganic systems force field (ff99SB) was applied to depict CpomPBP1 protein parameters^39^. An appropriate number of counterions were added to ensure the entire system at pH 7.0. For CpomPBP1-ETrME complex, we performed 75 ns MD simulations for production phase without any restraint. MD results were analyzed with Ambertools13 package based on the 75 ns MD trajectories.

In order to estimate the contribution of CpomPBP1 residues to ETrME binding, the CpomPBP1-ETrME interaction spectrum was decomposed based on a per-residue method using the Molecular Mechanics-Poisson-Boltzmann Surface Area (MM-PBSA) decomposition analysis by the mmpbsa.py module in AMBER12^40^,^41^. The computational alanine scanning mutagenesis (ASM) protocol which is effective and reliable in predicting key residues in protein participated interactions were also performed^42^,^43^. The binding free energies for complexes formed by ETrME and CpomPBP1 proteins (wild and mutant types of CpomPBP1) were calculated using the MM-PBSA method^43^. All the detail of molecular docking, MD and ASM were performed according to former reports^19^,^44^,^45^.

### Site-directed mutagenesis and protein expression

According to the results of ASM, 5 sites were subjected to biologically site-directed mutagenesis. To generate mutant types of CpomPBP1, the method of overlap extension PCR was performed in the course of our study. For each mutant site, 20–30 nucleotides long sense and antisense primers containing the target site were designed. Notably, the two primers of each site had better overlap more than 10 bp. Take Phe36 for example, three rounds PCR were required to change it into Ala. The first round PCR was conducted by taking P1F and F36Rm, P1R and F36Fm each as primer pairs. In the second round, 5–10 cycles of PCR were performed with the former two products as primers and templates each other. Thereafter, the mutant types of CpomPBP1 gene were obtained by taking PCR products of the second round as templates and P1F/P1R as primer pairs, and confirmed by gene sequencing. The rest mutants were prepared in the same way.

The 5 mutant types (MT) and wild type (WT) of CpomPBP1 genes were cloned into pET-28a (+) and transformed into competent Rosetta gami 2 cells. After 20 hrs induction with 0.6 mM IPTG under the condition of 16 °C 160 rpm, cultures expressing MT and WT proteins were collected by centrifugation (6000 rpm, 10 min) and broken by osmotic shock (10 s operation/10 s stop, 5 min). The periplasmic fractions were centrifuged at 12000 rpm for 30 min, the obtained supernatants were then loaded onto Ni^2+^-NTA sepharose gel column (7 Sea) and eluted according to the manufacturer’s manual. The purified proteins were analyzed by SDS-PAGE and dialyzed against 10 mM PBS (pH 7.4) before being quantified and kept in −20 °C for later binding assays.

To test binding ability changes of CpomPBP1 caused by the mutation of each target site, the *in vitro* binding assays between CpomPBP1 proteins (MT and WT CpomPBP1) and ETrME was implemented according to the part of competitive binding assay.

### Data analysis

It was assumed that CpomPBP1 (MT and WT) was 100% active and the binding between protein and ligand was 1:1 at saturation. To analyze the obtained data and to calculate the *K*_d_ of each ligand, Graphpad Prism software (Graphpad software, Inc.) was employed.

The experimental binding free energy of the complexes formed by ETrME and each mutant was calculated by the equation (1):


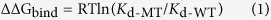


In this equation, *K*_d-MT_ and *K*_d-WT_ are the dissociation constants for mutant and wild CpomPBP1 respectively. R stands for the ideal gas constant and T means the temperature in Kelvin.

## Additional Information

**How to cite this article**: Liu, J. *et al.* Structure-based discovery of potentially active semiochemicals for *Cydia pomonella* (L.). *Sci. Rep.* **6**, 34600; doi: 10.1038/srep34600 (2016).

## Supplementary Material

Supplementary Information

## Figures and Tables

**Table 1:** The theoretical and experimental ΔΔG_bind_ avalues for wild and mutant CpomPBP1-ETrME complexes.

Protein^c^	S9A	F12A	F33A	F36A	W37A	I52A	S56A	I94A
ΔΔG_bind-cal_	−0.56	4.13	−2.13	4.55	5.21	2.31	−0.08	3.23
ΔΔG_bind-exp_^b^	—	2.80	—	3.25	3.54	1.86	—	1.4

^a^All values are given in kcal/mol, theoretical and experimental ΔΔG_bind_ are written as ΔΔG_bind-cal_ and ΔΔG_bind-exp_.

^b^The binding free energy difference (ΔΔG_bind_) between the mutant and wild type complexes is defined as ΔΔG_bind_ = RTln(*K*_d-MT_/*K*_d-WT_), where R is the ideal gas constant and T is the temperature in K, *K*_d-MT_ and *K*_d-WT_ are dissociation constants for mutant and wild CpomPBP1-ETrME complexes, respectively.

^c^S9A, F12A, F33A, F36A, W37A, I52A, S56A and I94A are abbreviations for CpomPBP1S9A, CpomPBP1F12A, CpomPBP1F33A, CpomPBP1F36A, CpomPBP1W37A, CpomPBP1I52A, CpomPBP1S56A and CpomPBP1I94A respectively.
