# Supplementary material for: Structure-based discovery of potentially active semiochemicals for Cydia pomonella (L.)
Source: Sci Rep. 2016 Oct 6;6:34600. doi: 10.1038/srep34600 (PMC5052595; doi:10.1038/srep34600)
Supplement: Supplementary Information [file srep34600-s1.pdf]

Structure-based discovery of potentially active semiochemicals for *Cydia pomonella* (L.)

Jiyuan Liu[1,2a]   Zhen Tian[1a]   Yalin Zhang[1]*

[1] Key Laboratory of Plant Protection Resources & Pest Management of the Ministry of Education, College of Plant Protection, Northwest A&F University, Yangling 712100, Shaanxi, China

[2] Department of Medicinal Chemistry, School of Pharmacy, Fourth Military Medical University, Xi’an 710032, Shaanxi, China

[a] These authors contributed equally to this work.

*Corresponding author: Ya-Lin Zhang

Key Laboratory of Plant Protection Resources & Pest Management of the Ministry of Education, Northwest A&F University, Yangling 712100, Shaanxi, China. Tel./Fax: +86-29-8709-2190. E-mail: yalinzh@nwsuaf.edu.cn.

Preparations for structure-based pharmacophore modeling

Details for 3D model construction The 3D structure of CpomPBP1 was built by using Modeller9.10[1]. Based on the crystallographic R-factor (21.8%), sequential identity (50%, Figure S1b) and pH state (pH 7.0), we finally selected the crystal structure of BmorPBP-bombykol complex from *Bombyx mori* (PDB ID: 1DQE, Chain A, resolution 1.8 Å) as the template for homology modelling. The CpomPBP1-Codlemone and CpomPBP1-ETrME complexes were constructed by molecular docking simulations using program GOLD5.3[2]. The 3D model of CpomPBP1 was performed 5000 steps minimization in Amber12 with ff99SB force field[3] before being selected as a receptor for docking simulations. The 3D structure of Codlemone was sketched using Maestro version (Schrodinger Inc.), and the 3D structure of ETrME was derived from the results of virtual screening proceeded by Ligandsout4.09[4]. Whole of them were optimized 2000 steps in Amber12 with GAFF force field[5] prior to the docking simulation. The Chemplp score implemented in GOLD was employed to finely reproduce the best binding model of these complexes since it is superior to other scoring functions in GOLD for pose prediction[6]. All details of the homology modeling and the molecular docking were performed according our previous reports[7,8].

Protonation states examination of CpomPBP1 model The protonation states of CpomPBP1 model were examined by using Rosetta pKa Protocol[9] before we loaded the homology model of CpomPBP1 into the GOLD5.3 for molecule docking. Based on the CpomPBP1 model, we estimated the pKa values of five types of residues (Asp, Glu, His, Tyr, and Lys) in varied protonation states (Table S8). These ionizable residues, especially His35, His69, His70, His80, His95 and His123, were expected to show smaller pKa shifts compared to their theoretical intrinsic pKa (IpKa) values (set protonation with proton on Nδ1 atoms). Moreover, According to the 2D interaction diagram of CpomPBP1 with Codlemone (Figure S10), these ionizable residues were far away from Codlemone binding sites. As a result of what mentioned above，the protonation states of CpomPBP1shed little effects on the structure-based pharmacophore modeling in this study。

Solvent selection for competitive binding assay

Parts of our tested compounds are stored in ethanol (GC grade) solution, so it is necessary to check whether ethanol is advisable to be the solvent in competitive binding assay. Two

experiments were performed to evaluate the effects of ethanol on CpomPBP1 protein and CpomPBP1/1-NPN system. One was titrating 2 μM CpomPBP1 solution with ethanol (final volume fraction ranging from 0 to 50%), This experiment suggested the effects of ethanol on CpomPBP1 protein. The other was titrating solutions containing 2 μM CpomPBP1 and 2 μM 1-NPN with ethanol (0-32 μM). In the pretests, the fluorescence intensity at each point was recorded by Hitachi F-4600 spectrofluorimeter with a slit width of 5 nm for excitation and emission. For the first experiment, the excitation and emission wavelength were determined to be 250 nm and 280-380nm, respectively. For the second experiment, the excitation and emission wavelength were determined to be 370 nm and 385-500nm, respectively.

Supplementary Tables

Table S1 Pharmacophore-Fit Score obtained through virtual screening and the extracted 133 hits.

Entry	Zinc No.	Source Database	Pharmacophore-Fit Score
1	ZINC39381666	semiochemicals.ldb	47.71
2	ZINC13508540	semiochemicals.ldb	47.52
3	ZINC83314692	semiochemicals.ldb	47.51
4	ZINC83314700	semiochemicals.ldb	47.50
5	ZINC83314696	semiochemicals.ldb	47.48
6	ZINC83314697	semiochemicals.ldb	47.26
7	ZINC04521470	semiochemicals.ldb	47.24
8	ZINC27643568	semiochemicals.ldb	47.21
9	ZINC83316818	semiochemicals.ldb	47.20
10	ZINC31261530	semiochemicals.ldb	47.19
11	ZINC12496534	semiochemicals.ldb	47.19
12	ZINC12495672	semiochemicals.ldb	47.19
13	ZINC27646426	semiochemicals.ldb	47.18
14	ZINC64858257	semiochemicals.ldb	47.15
15	ZINC08860491	semiochemicals.ldb	47.11
16	ZINC64220083	semiochemicals.ldb	47.10
17	ZINC13507101	semiochemicals.ldb	47.10
18	ZINC12495497	semiochemicals.ldb	47.10
19	ZINC33820499	semiochemicals.ldb	47.07
20	ZINC83314695	semiochemicals.ldb	47.06
21	ZINC27643574	semiochemicals.ldb	47.06
22	ZINC27645640	semiochemicals.ldb	47.03
23	ZINC04543843	semiochemicals.ldb	47.03
24	ZINC03630789	semiochemicals.ldb	47.02
25	ZINC33822158	semiochemicals.ldb	46.98
26	ZINC33954700	semiochemicals.ldb	46.96
27	ZINC27643447	semiochemicals.ldb	46.96
28	ZINC64219875	semiochemicals.ldb	46.95
29	ZINC12495502	semiochemicals.ldb	46.93
30	ZINC04655401	semiochemicals.ldb	46.93
31	ZINC64859078	semiochemicals.ldb	46.9
32	ZINC13508536	semiochemicals.ldb	46.89
33	ZINC90427610	semiochemicals.ldb	46.88
34	ZINC33822158	semiochemicals.ldb	46.88
35	ZINC40164570	semiochemicals.ldb	46.86
36	ZINC34961834	semiochemicals.ldb	46.86
37	ZINC90427607	semiochemicals.ldb	46.85
38	ZINC27643454	semiochemicals.ldb	46.85
39	ZINC27644605	semiochemicals.ldb	46.82
40	ZINC04544257	semiochemicals.ldb	46.80

41	ZINC03802189	semiochemicals.ldb	46.78
42	ZINC26578389	semiochemicals.ldb	46.74
43	ZINC64858252	semiochemicals.ldb	46.72
44	ZINC44608726	semiochemicals.ldb	46.72
45	ZINC02139103	semiochemicals.ldb	46.72
46	ZINC31333928	semiochemicals.ldb	46.70
47	ZINC08860470	semiochemicals.ldb	46.69
48	ZINC31983243	semiochemicals.ldb	46.68
49	ZINC64219883	semiochemicals.ldb	46.67
50	ZINC04543724	semiochemicals.ldb	46.67
51	ZINC64858254	semiochemicals.ldb	46.66
52	ZINC02040077	semiochemicals.ldb	46.65
53	ZINC64219881	semiochemicals.ldb	46.59
54	ZINC83316820	semiochemicals.ldb	46.58
55	ZINC14984556	semiochemicals.ldb	46.58
56	ZINC30730667	semiochemicals.ldb	46.51
57	ZINC27644415	semiochemicals.ldb	46.51
58	ZINC27643501	semiochemicals.ldb	46.49
59	ZINC83314732	semiochemicals.ldb	46.47
60	ZINC64219882	semiochemicals.ldb	46.46
61	ZINC03777423	semiochemicals.ldb	46.46
62	ZINC05761860	semiochemicals.ldb	46.45
63	ZINC64219870	semiochemicals.ldb	46.44
64	ZINC83316817	semiochemicals.ldb	46.41
65	ZINC31261534	semiochemicals.ldb	46.41
66	ZINC27643561	semiochemicals.ldb	46.40
67	ZINC14494726	semiochemicals.ldb	46.40
68	ZINC12504490	semiochemicals.ldb	46.40
69	ZINC04655397	semiochemicals.ldb	46.39
70	ZINC14616296	semiochemicals.ldb	46.38
71	ZINC90372639	semiochemicals.ldb	46.37
72	ZINC33953561	semiochemicals.ldb	46.36
73	ZINC04655400	semiochemicals.ldb	46.35
74	ZINC64220091	semiochemicals.ldb	46.34
75	ZINC13543118	semiochemicals.ldb	46.34
76	ZINC12496042	semiochemicals.ldb	46.34
77	ZINC90372641	semiochemicals.ldb	46.32
78	ZINC33822144	semiochemicals.ldb	46.28
79	ZINC15273980	semiochemicals.ldb	46.24
80	ZINC04528924	semiochemicals.ldb	46.22
81	ZINC90372638	semiochemicals.ldb	46.19
82	ZINC30730644	semiochemicals.ldb	46.19
83	ZINC12496917	semiochemicals.ldb	46.16
84	ZINC26578301	semiochemicals.ldb	46.14

85	ZINC90427606	semiochemicals.ldb	46.13
86	ZINC83314698	semiochemicals.ldb	46.13
87	ZINC05964866	semiochemicals.ldb	46.13
88	ZINC27643554	semiochemicals.ldb	46.12
89	ZINC08860439	semiochemicals.ldb	46.12
90	ZINC03871295	semiochemicals.ldb	46.12
91	ZINC64219873	semiochemicals.ldb	46.11
92	ZINC13516216	semiochemicals.ldb	46.09
93	ZINC64219888	semiochemicals.ldb	46.07
94	ZINC31333933	semiochemicals.ldb	46.07
95	ZINC27643443	semiochemicals.ldb	46.07
96	ZINC33838356	semiochemicals.ldb	46.02
97	ZINC27643057	semiochemicals.ldb	46.01
98	ZINC83314702	semiochemicals.ldb	45.97
99	ZINC64858265	semiochemicals.ldb	45.97
100	ZINC13543119	semiochemicals.ldb	45.94
101	ZINC27643481	semiochemicals.ldb	45.91
102	ZINC83316566	semiochemicals.ldb	45.89
103	ZINC14616300	semiochemicals.ldb	45.89
104	ZINC08860471	semiochemicals.ldb	45.88
105	ZINC27643488	semiochemicals.ldb	45.82
106	ZINC13508538	semiochemicals.ldb	45.81
107	ZINC83316749	semiochemicals.ldb	45.8
108	ZINC04474613	semiochemicals.ldb	45.78
109	ZINC13507528	semiochemicals.ldb	45.77
110	ZINC27643494	semiochemicals.ldb	45.74
111	ZINC34961865	semiochemicals.ldb	45.72
112	ZINC34022433	semiochemicals.ldb	45.72
113	ZINC13551720	semiochemicals.ldb	45.7
114	ZINC05510392	semiochemicals.ldb	45.69
115	ZINC83314689	semiochemicals.ldb	45.64
116	ZINC05417126	semiochemicals.ldb	45.64
117	ZINC33953352	semiochemicals.ldb	45.63
118	ZINC14494723	semiochemicals.ldb	45.61
119	ZINC05510380	semiochemicals.ldb	45.59
120	ZINC90372640	semiochemicals.ldb	45.58
121	ZINC59383589	semiochemicals.ldb	45.56
122	ZINC33822120	semiochemicals.ldb	45.56
123	ZINC04556502	semiochemicals.ldb	45.54
124	ZINC05510396	semiochemicals.ldb	45.53
125	ZINC08860440	semiochemicals.ldb	45.47
126	ZINC12493948	semiochemicals.ldb	45.42
127	ZINC83314729	semiochemicals.ldb	45.37
128	ZINC33820296	semiochemicals.ldb	45.37

129	ZINC70776987	semiochemicals.ldb	45.27
130	ZINC70774858	semiochemicals.ldb	45.20
131	ZINC33611444	semiochemicals.ldb	44.71
132	ZINC33611443	semiochemicals.ldb	44.69
133	ZINC33611445	semiochemicals.ldb	44.68

Table S2 Gaussian Shape Similarity Score obtained based on shape alignment and the extracted 31 hits.

Entry	Zinc No.	Pharmacophore-Fit Score	Gaussian Shape Similarity Score
1	ZINC83316566	45.89	0.67234
2	ZINC83314700	47.5	0.64463
3	ZINC15273980	46.24	0.63166
4	ZINC27643501	46.49	0.62931
5	ZINC04655401	46.93	0.62170
6	ZINC83314697	46.64	0.62103
7	ZINC03630789	47.02	0.62072
8	ZINC83316818	47.2	0.60465
9	ZINC27643568	47.21	0.60282
10	ZINC27643488	45.72	0.59936
11	ZINC13508540	47.52	0.59149
12	ZINC83314696	47.35	0.58634
13	ZINC34961865	45.56	0.58551
14	ZINC05964866	46.13	0.58093
15	ZINC04474613	45.78	0.57441
16	ZINC03871295	46.12	0.56778
17	ZINC64858254	46.35	0.56712
18	ZINC27643554	46.12	0.56462
19	ZINC83314695	46.76	0.55472
20	ZINC83316817	46.32	0.55096
21	ZINC40164570	46.86	0.54915
22	ZINC13508536	46.89	0.54795
23	ZINC31333928	46.7	0.54648
24	ZINC83314698	46.13	0.54516
25	ZINC33822158	46.88	0.54419
26	ZINC04655400	46.35	0.54101
27	ZINC13551720	45.7	0.53500
28	ZINC31983243	46.68	0.53477
29	ZINC12496534	47.19	0.52698
30	ZINC13507101	47.1	0.52582
31	ZINC30730667	46.51	0.52548

Table S3 Binding affinity calculation results and the extracted 18 hits.

Entry	Name	Gaussian Shape Similarity Score	Pharmacophore-Fit Score	Binding Affinity Score
1	ZINC13508536	0.54795	46.89	-35.99
2	ZINC31983243	0.53477	46.68	-35.17
3	ZINC83314697	0.62103	46.64	-35.05
4	ZINC27643501	0.62931	46.49	-35.00
5	ZINC13551720	0.535	45.7	-34.83
6	ZINC03630789	0.62072	47.02	-34.62
7	ZINC33822158	0.54419	46.88	-34.49
8	ZINC83314698	0.54516	46.13	-34.29
9	ZINC30730667	0.52548	46.51	-34.18
10	ZINC83316818	0.60465	47.2	-33.65
11	ZINC13508540	0.59149	47.52	-33.48
12	ZINC13507101	0.52582	47.1	-33.48
13	ZINC83314700	0.56307	46.15	-33.40
14	ZINC83316566	0.67234	45.89	-32.95
15	ZINC83316817	0.55096	46.32	-32.90
16	ZINC04474613	0.57441	45.78	-32.52
17	ZINC83314696	0.58634	47.35	-32.46
18	ZINC12496534	0.52698	47.19	-30.79

Table S4 Physical properties prediction of 4 hit compounds and Codlemone.

Compound	Physical state	Boiling Point [a]	Vapor Pressure [b]	Enthalpy of Vaporization [c] (kcal/mol)
ETrME	liquid	398.55±11.00	-5.84 ±0.92	15.51
Codlemone	liquid	270.68±9.00	-3.05 ±1.24	14.12
ZINC27643501	liquid	388.18±11.00	-6.38 ±1.90	16.72
ZINC12496534	-	488.04±45.00	0.00 ±2.80	20.76
ZINC13507101	-	437.98±24.00	-8.17 ±2.26	18.21

[a] Calculated boiling point value at 101.325 kPa pressure.

[b] Saturated vapor pressure prediction of compounds in the closed equilibrium system at the temperature of 25 Celsius. Vapor pressure prediction is converted into a logarithmic scale.

[c] Calculated enthalpy of vaporization indicates the amount of energy needed to vaporize a given quantity of analyzed compound under the atmospheric pressure.

Table S5 Cluster analysis of CpomPBP1-ETrME complex based on the MD simulations trajectory[a].

Cluster	Occurrence [%]	RMSD [Å]
		To Docking
1	2.3	1.40
2(	6.2	1.25
3	6.5	1.33
4	37.1	1.23
5	47.8	1.13

[a] The five structural clusters appear consecutively during the 75 ns MD simulations. RMSD represents the conformational variations between clusters and docking structure.

Table S6 Decomposition of binding free energy on a per-residue level [a]

**Residue**	S_{VDW}	B_{VDW}	T_{VDW}	S_{ELE}	B_{ELE}	T_{ELE}	S_{EPB}	B_{EPB}	T_{EPB}	S_{TOT}	B_{TOT}	T_{TOT}
Phe12	-2.418	-0.218	-2.636	-0.124	-0.012	-0.136	1.228	0.030	1.259	-1.313	-0.200	-1.513
Phe36	-1.922	-0.204	-2.126	-0.128	-0.011	-0.139	1.007	-0.031	0.976	-1.043	-0.247	-1.289
Trp37	-1.481	-0.104	-1.584	-1.614	0.002	-1.612	1.638	0.103	1.741	-1.456	0.001	-1.455
Ile52	-1.138	-0.272	-1.409	0.040	-0.042	-0.002	0.084	0.407	0.491	-1.014	0.093	-0.920
Ile94	-1.274	-0.190	-1.465	-0.019	-0.019	-0.038	0.013	0.086	0.099	-1.280	-0.124	-1.404
Ser9	-0.634	-0.853	-1.488	-0.117	-0.186	-0.303	1.256	0.712	1.968	1.256	-0.327	0.177
Phe33	-0.914	-0.194	-1.11	0.050	-0.084	-0.034	0.808	1.122	1.930	-0.057	0.844	0.787
Ser56	-0.472	-0.157	-0.629	-0.036	0.011	-0.024	1.907	-0.000	1.907	1.400	-0.147	1.254

[a] Energies shown as contributions of sidechain atoms (S), backbone atoms (B), and the total (T). Each item is subdivided into van der Waals energy (VDW), electrostatic energy (ELE), polar solvation energy (EPB). TOT represents the total energy contribution of each item. All values are given in kcal/mol.

Table S7 H-bond interactions between ETrME and CpomPBP1 during whole MD simulations[a].

DONOR		ACCEPTORH		ACCEPTOR				
atom#	:res@atom	atom#	:res@atom	atom#	:res@atom	%occupied	distance	angle
2302	:146@O21	567	:37@HE1	566	:37@NE1	74.84	2.93 (0.17)	30.72 (12.85)

[a] The percentage of simulation snapshots (saved every 10 p sec) in which the H-bond was present is listed. The occupancy of H-bonds larger than 5% is listed.

Table S8 Calculation of pKa values for all ionizable residues[a] in CpomPBP1 structure.

Residue Number	IpKa[b]	pKa	Method [c]
GLU 2	4.4	5.7	N
ASP 7	4	3.9	N
LYS 14	10.4	10.5	N
ASP 17	4	3.9	N
GLU 18	4.4	3.8	N
LYS 20	10.4	10.9	N
LYS 21	10.4	10.4	N
ASP 22	4	4	N
GLU 23	4.4	4.6	N
ASP 27	4	3.1	N
ASP 32	4	1	N
HIS 35	6.3	6	N
LYS 38	10.4	10.3	N
GLU 39	4.4	4.4	N
ASP 40	4	3.6	N
TYR 41	10	10	N
LYS 57	10.4	10.3	N
LYS 58	10.4	11.8	N
GLU 60	4.4	4.6	N
ASP 63	4	3.3	N
ASP 65	4	5.1	N
GLU 67	4.4	5.1	N
HIS 69	6.3	4.3	N
HIS 70	6.3	5.9	N
LYS 74	10.4	10	N
GLU 75	4.4	4.8	N
LYS 79	10.4	10.4	N
HIS 80	6.3	5	P
ASP 83	4	3	N
GLU 84	4.4	4.4	N
GLU 85	4.4	5.6	N
ASP 89	4	4.1	N
HIS 95	6.3	6.4	N
ASP 96	4	3.7	N
ASP 105	4	3.7	N
ASP 106	4	2.9	N
LYS 110	10.4	10	N
GLU 113	4.4	3.8	N
LYS 116	10.6	11.3	N
LYS 119	10.4	11.4	N
GLU 121	4.4	4	N
HIS 123	6.3	6.1	N

LYS 124	10.4	11	N
LYS 125	10.4	10.5	N
GLU 132	4.4	4.5	N
GLU 137	4.4	4.3	N
GLU 141	4.4	5.1	N

[a] All ionizable residues in CpomPBP1 were categorized into five types of Asp, Glu, His, Tyr and Lys.

[b] IpKa represents the theoretical intrinsic pKa values. Ionizable residues buried in the core of the protein and near enzyme catalytic sites are expected to show higher pKa shifts compared to the residues on the protein surface exposed to the solvent.

[c] Methods: S: Site Repack; N: Neighbor Repack; P: Prepack Complete Structure.

Supplementary Figure Legends

Figure S1 Structure of CpomPBP1. (a) 3D model of CpomPBP1. The six α-helixes are marked by α1 to α6, C and N represent C-terminus and N-terminus, respectively. (b) Amino acid sequences alignment between BmorPBP from *Bombyx mori* and CpomPBP1. 1DQE_A stands for BmorPBP.

Figure S2 Interaction energy map between CpomPBP1 (in the binding site region) and probe. The golden (a) and slate blue (b) points indicate areas of favorable interaction energy with hydroxyl and CMET probes (energy threshold set to -18.9 and 18.1KJ/mol, respectively). Codlemone was overlaid for comparison, but removed before computing the interaction energy map.

Figure S3 The effects of ethanol on CpomPBP1 (a) and CpomPBP1-1-NPN system (b). F_0 is the intrinsic fluorescence intensity of CpomPBP1 without ethanol, F_m is the intrinsic fluorescence intensity of CpomPBP1 at a given ethanol concentration.

Figure S4 Binding curves of tested ligands to CpomPBP1. These ligands including C20:3n-6,8,12 (a), OTrE (b) and Linoleic acid (c) exhibited no binding to CpomPBP1.

Figure S5 Molecular dynamic analysis of CpomPBP1-ETrME complex. (a) RMSD value for the whole backbone of atoms from CpomPBP1-ETrME complex, (b) RMSD of ETrME relative to the starting structure during the production phase of MD simulations. (c) Average fluctuations of residues from CpomPBP1 in the whole process of 75 ns MD simulations.

Figure S6 Superimposing the best MD representative conformations of ETrME and key residues in the active pocket of CpomPBP1 on the counterparts of docking structure. In green, the best MD representative conformation; in gray, the conformations of docking structure. ETrME is presented with stick-and-sphere model. Key residues including Phe12, Phe36 and Trp37 are presented with stick model. Red dashed line is the H bond.

Figure S7 The distances of key interactions in CpomPBP1-ETrME complex. (a) The best MD (molecular dynamic) representative structure of CpomPBP1-ETrME complex. ETrME and key residues including Phe12, Phe36 and Trp37 are presented in green and gray stick-and-sphere model, respectively. Black, red and green dashed lines represent the average distances of key interactions. (b) The distances of atoms forming key interactions along the whole MD simulation time. Corresponding atoms are marked in (a).

Figure S8 Residue-ligand interaction spectrum of the CpomPBP1-ETrME according to the MM-PBSA method. The x-axis denotes the residue number of CpomPBP1 and the y-axis denotes

the sidechain energy contribution of each residue.

Figure S9 Site-directed mutation of CpomPBP1. (a) The site-directed mutation of CpomPBP1 gene. (b) The mutant and wild types of CpomPBP1 protein. F12A, F36A, W37A, I52A and I94A are abbreviations for CpomPBP1F12A, CpomPBP1F36A, CpomPBP1W37A, CpomPBP1I52A and CpomPBP1I94A. WT stands for wildtype CpomPBP1.

Figure S10 The interaction diagram of CpomPBP1 with ETrME. Only van der waals (light green) and hydrogen bond (green) interactions were detected around the binding sites of CpomPBP1.

Figure S1

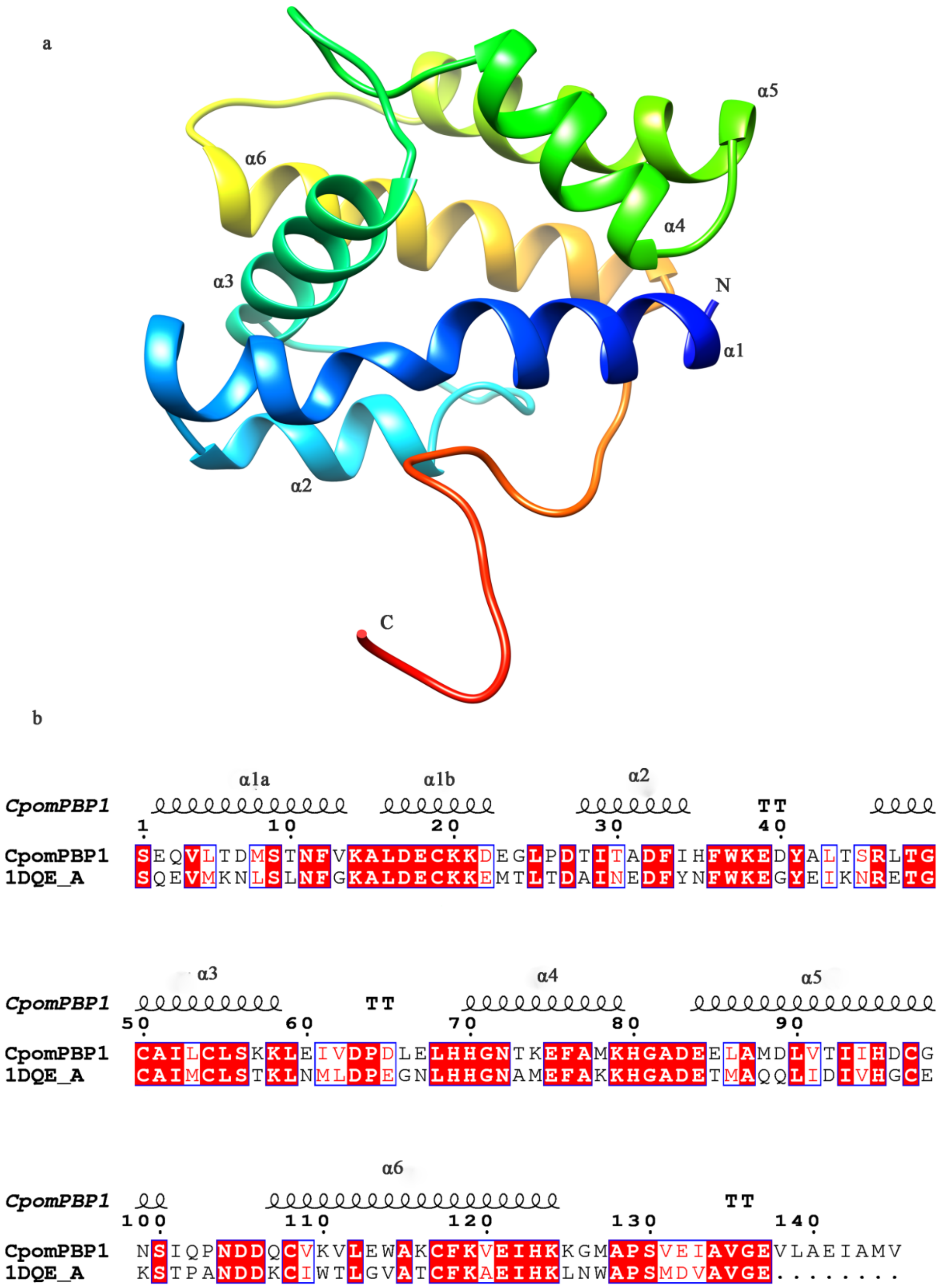
a
α5
α6
α4
α3
N
α1
α2
C
b
α1a
α1b
α2
CpomPBP1
TT
1
10
20
30
40
CpomPBP1 SEQVLTDMSTNFVKALDECKKDEGLPDTITADFIHFWKEDYALTSRLTG
1DQE_A SQEVMKNLSLNFGKALDECKKEMTLTDAINEDFYNFWKEGYEIKNRETG
α3
α4
α5
CpomPBP1
TT
50
60
70
80
90
CpomPBP1 CAILCLSKKLEIVDPDLELHHGNTKEFAMKHGADEELAMDLVTIIHDCG
1DQE_A CAIMCLSTKLNMLDPEGNLHHGNAMEFAKKHGADETMAQQLIDIVHGCE
α6
CpomPBP1
TT
100
110
120
130
140
CpomPBP1 NSIQPNDDQCVKVLEWAKCFKVEIHKKGMAPSVEIAVGEVLAEIAMV
1DQE_A KSTPANDDKCIWTLGVATCFKAEIHKLNWAPSMDVAVGE........

Figure S2

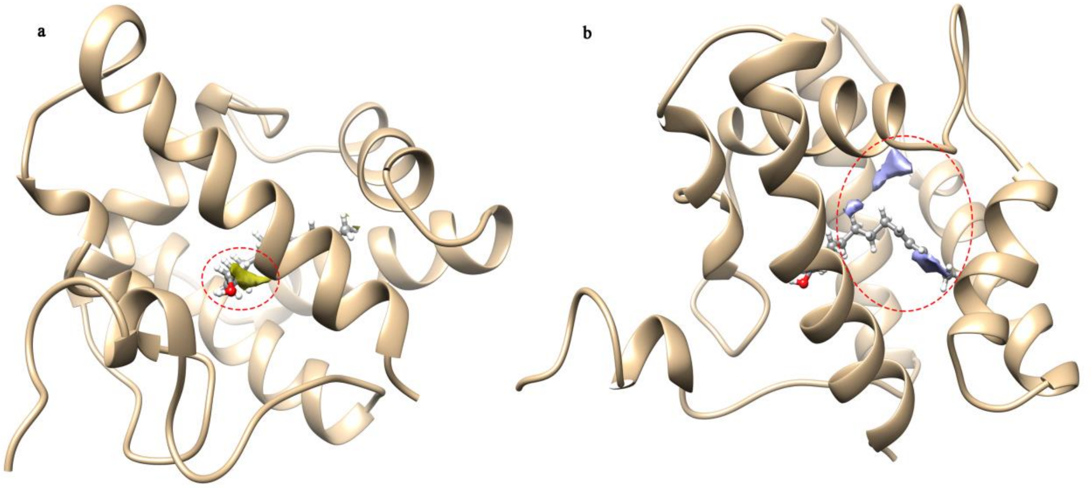

Figure S3

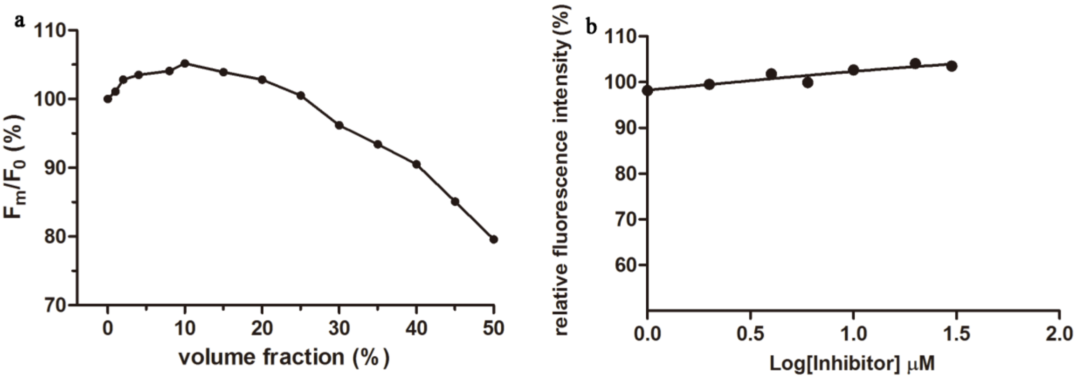

Figure S4

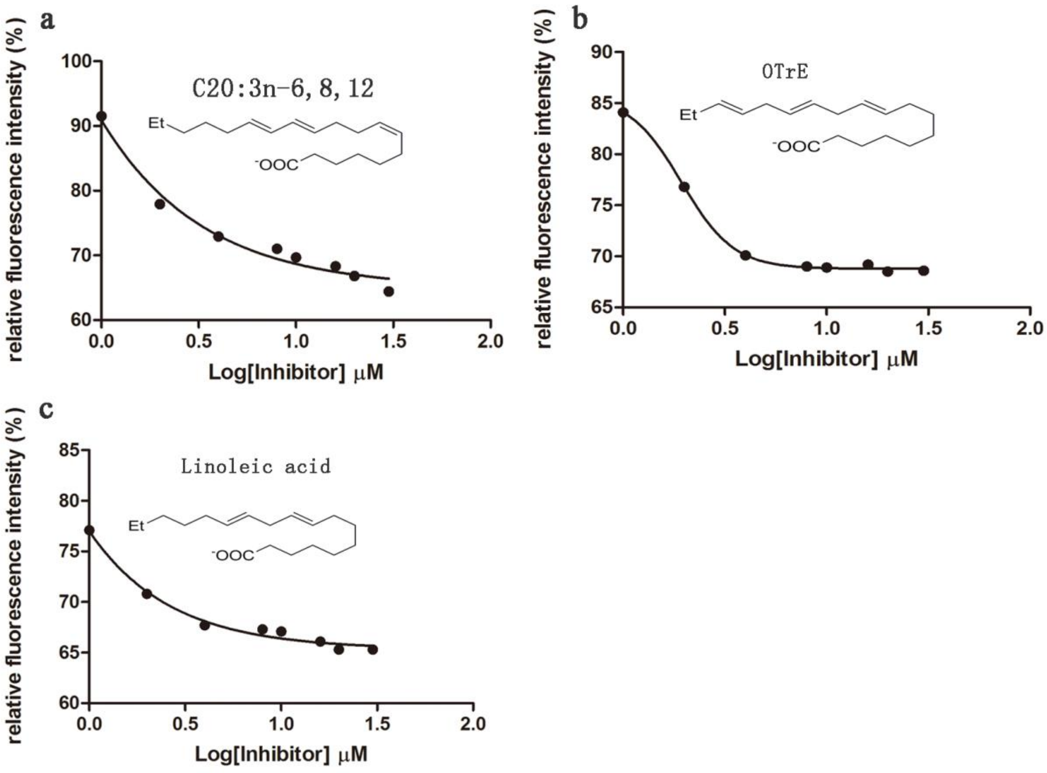
a
C20:3n-6,8,12
Et
-OOC
relative fluorescence intensity (%)
Log[Inhibitor] μM
b
OTrE
Et
-OOC
relative fluorescence intensity (%)
Log[Inhibitor] μM
c
Linoleic acid
Et
-OOC
relative fluorescence intensity (%)
Log[Inhibitor] μM

Figure S5

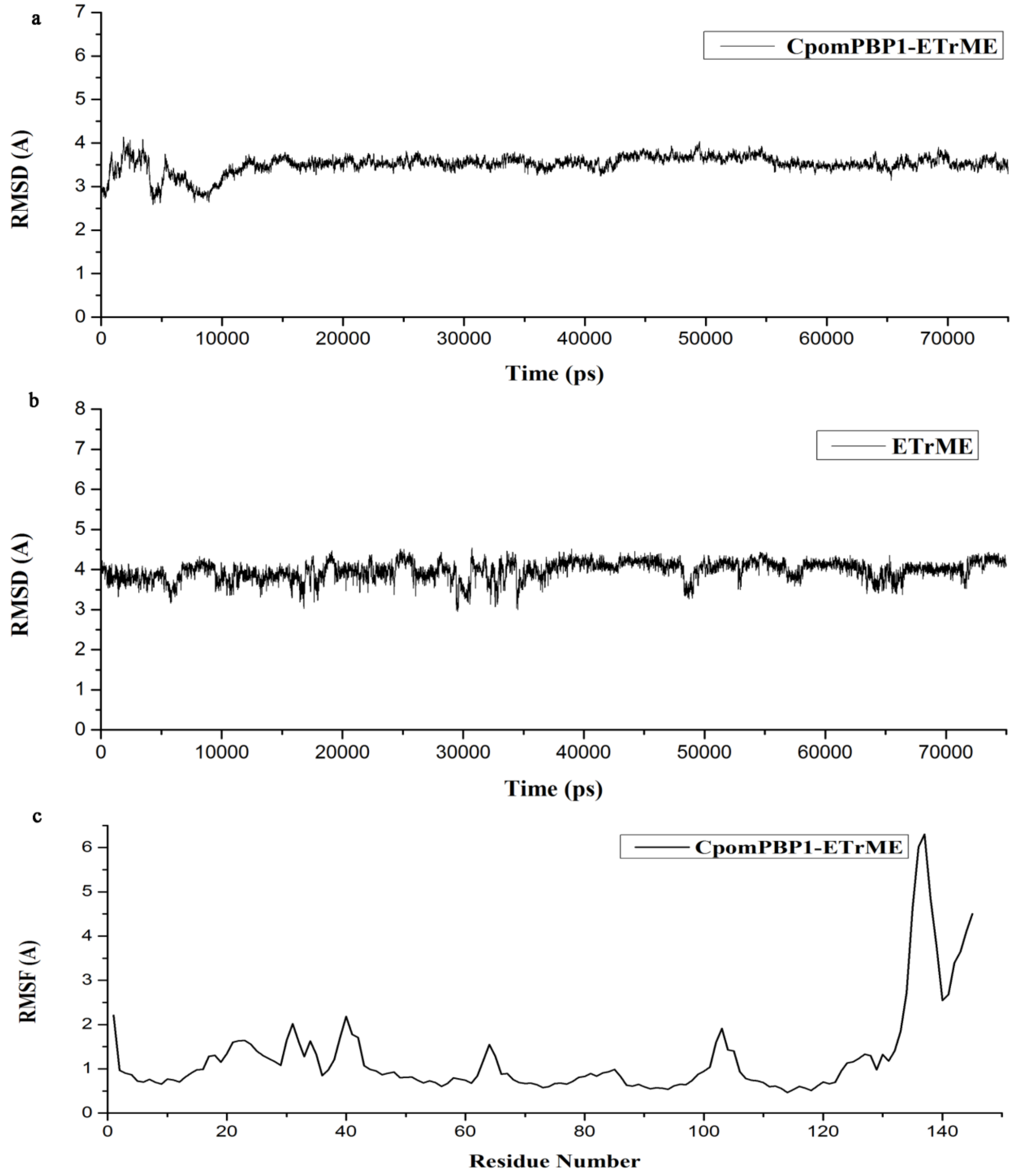
a
RMSD (A)
Time (ps)
CpomPBP1-ETrME
b
RMSD (A)
Time (ps)
ETrME
c
RMSF (A)
Residue Number
CpomPBP1-ETrME

Figure S6

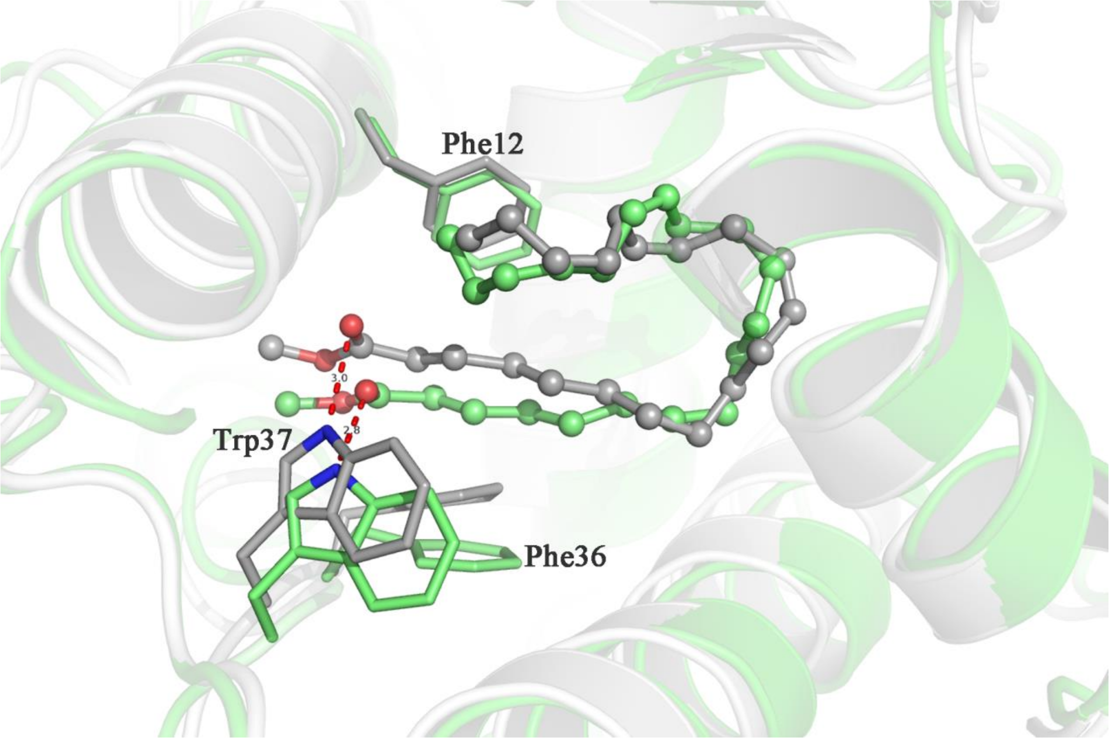

Figure S7

a

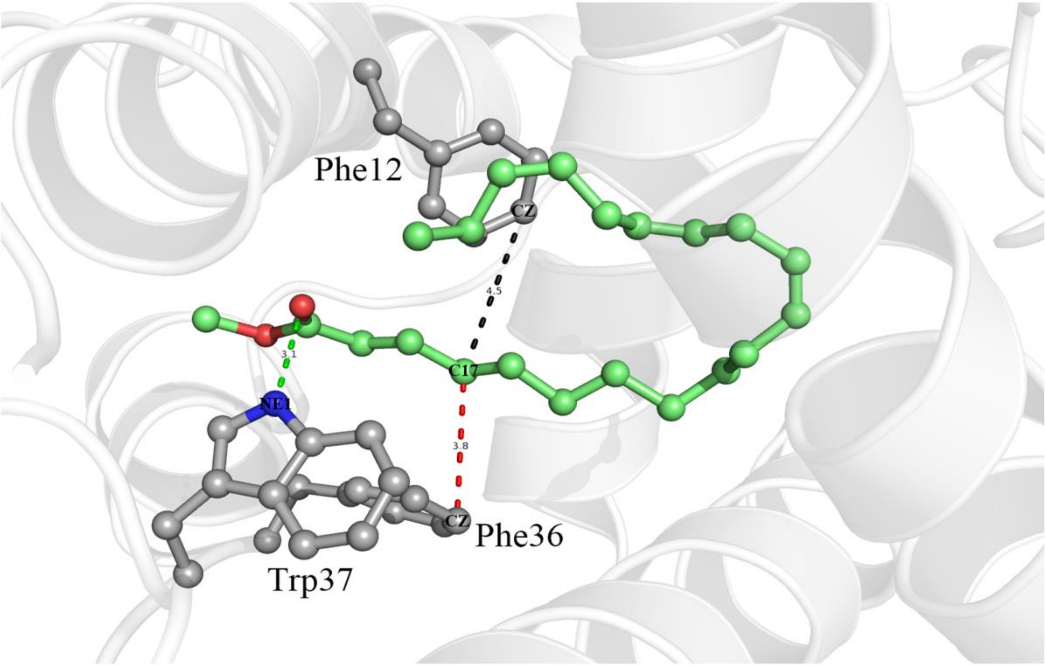
Phe12
CZ
4.5
3.1
NE1
C17
3.8
CZ
Phe36
Trp37

b

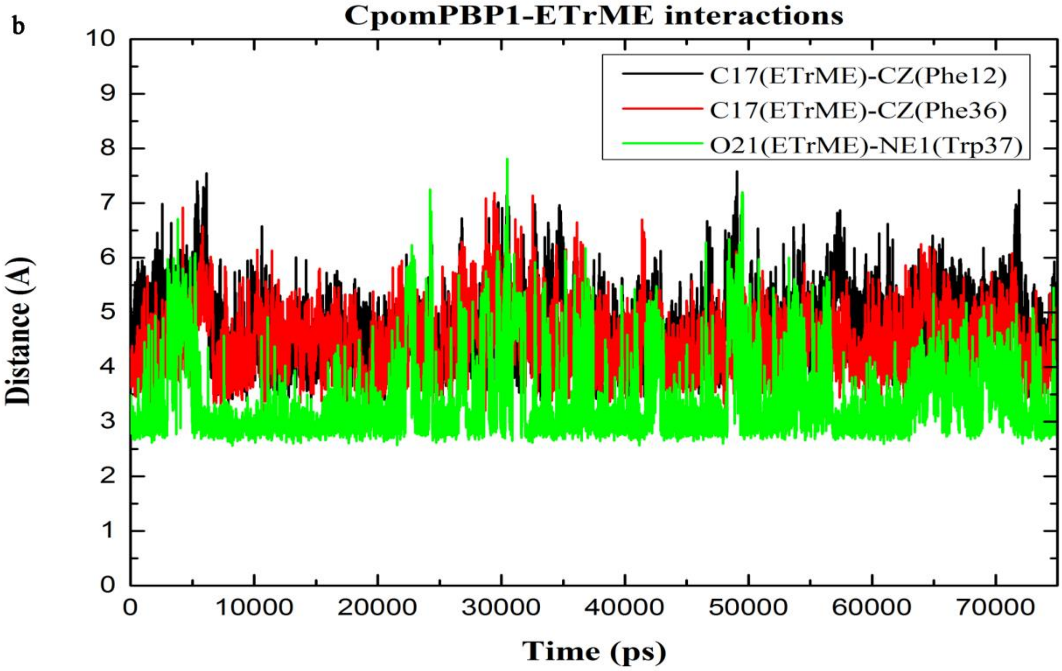
CpomPBP1-ETrME interactions
C17(ETrME)-CZ(Phe12)
C17(ETrME)-CZ(Phe36)
O21(ETrME)-NE1(Trp37)
Distance (A)
Time (ps)
0
1
2
3
4
5
6
7
8
9
10
0
10000
20000
30000
40000
50000
60000
70000

Figure S8

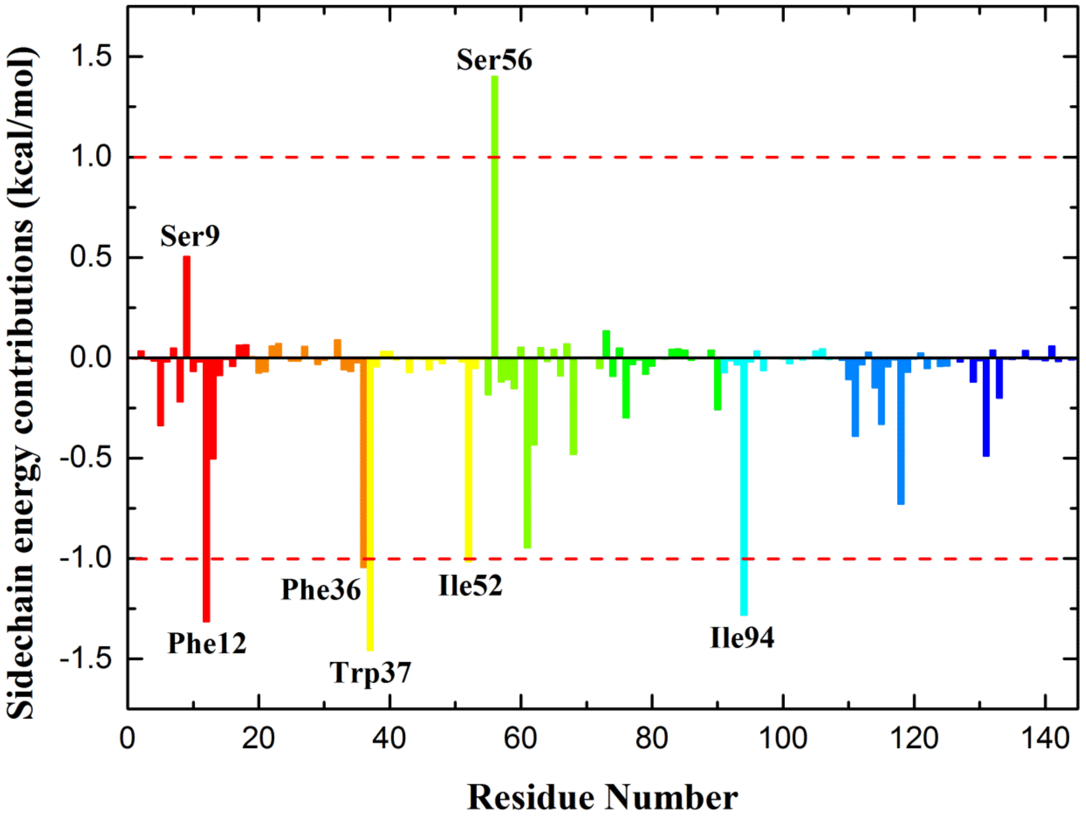

Sidechain energy contributions (kcal/mol)
1.5
1.0
0.5
0.0
-0.5
-1.0
-1.5
0
20
40
60
80
100
120
140
Residue Number
Ser56
Ser9
Phe36
Ile52
Phe12
Trp37
Ile94

Figure S9

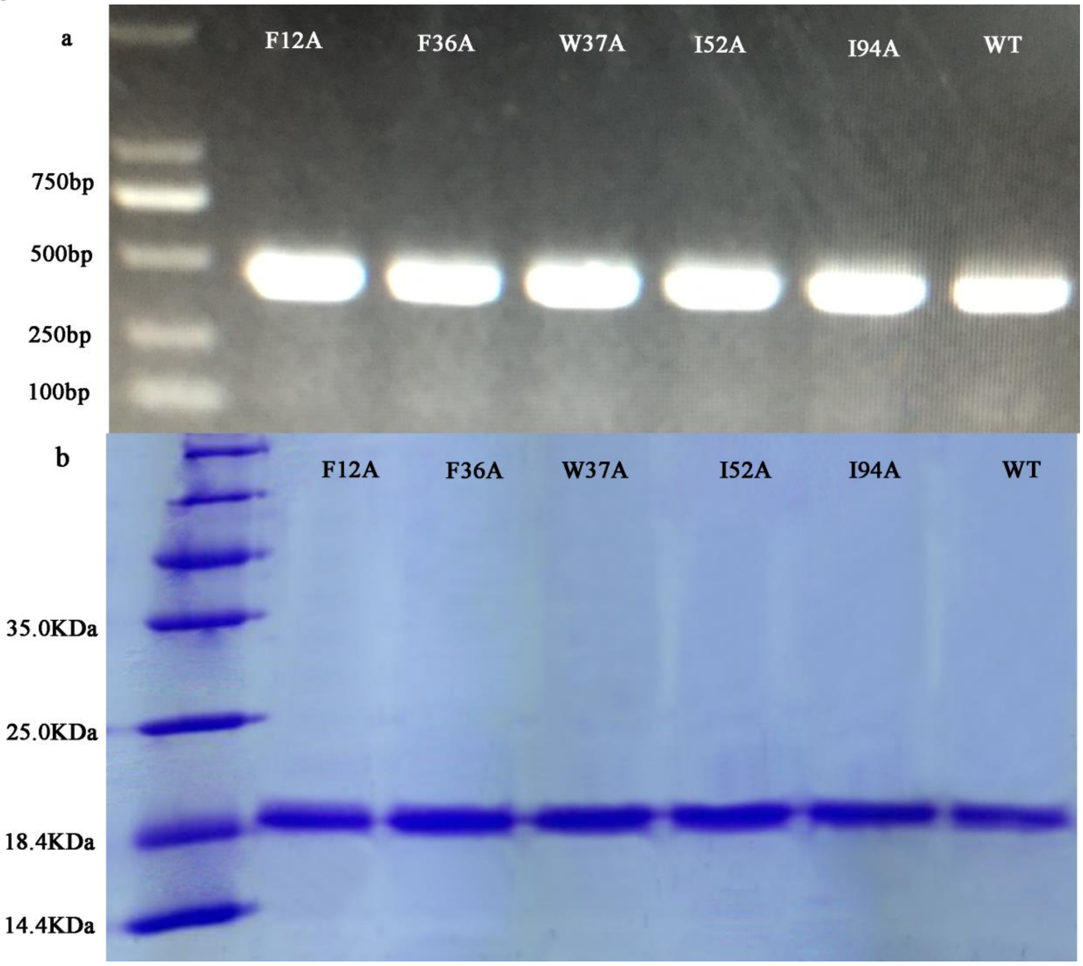
a
F12A
F36A
W37A
I52A
I94A
WT
750bp
500bp
250bp
100bp
b
F12A
F36A
W37A
I52A
I94A
WT
35.0KDa
25.0KDa
18.4KDa
14.4KDa

Figure S10

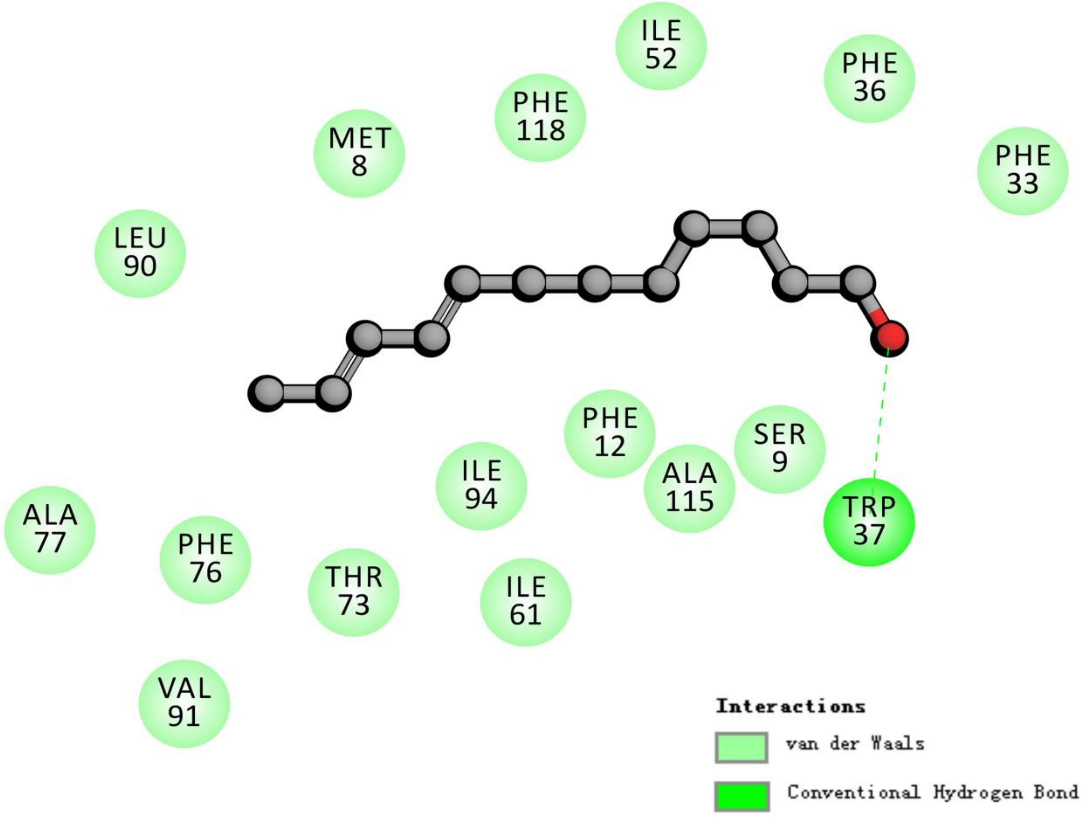
ILE 52
PHE 36
PHE 118
MET 8
PHE 33
LEU 90
PHE 12
SER 9
ILE 94
ALA 115
TRP 37
ALA 77
PHE 76
THR 73
ILE 61
VAL 91
Interactions
van der Waals
Conventional Hydrogen Bond

References

1 Šali & Blundell, T. L. Comparative protein modelling by satisfication of spatial restraints. *J Mol Biol* **234**, 779 (1993).

2 Jones, G., Willett, P., Glen, R. C., Leach, A. R. & Taylor, R. Development and validation of a genetic algorithm for flexible docking. *J Mol Biol* **267**, 727-748 (1997).

3 Hummer, G., Rasaiah, J. C. & Noworyta, J. P. Water conduction through the hydrophobic channel of a carbon nanotube. *Nature* **414**, 188-190 (2001).

4 Wolber, G. & Langer, T. LigandScout: 3-D pharmacophores derived from protein-bound ligands and their use as virtual screening filters. *J Chem Inf Model* **45**, 160-169 (2005).

5 Wang, J., Wolf, R. M., Caldwell, J. W., Kollman, P. A. & Case, D. A. Development and testing of a general amber force field. *J Comput Chem* **25**, 1157-1174 (2004).

6 Korb, O., Stutzle, T. & Exner, T. E. Empirical scoring functions for advanced protein−ligand docking with PLANTS. *J Chem Inf Model* **49**, 84-96 (2009).

7 Liu, J., Yang, X. & Zhang, Y. Characterization of a lambda-cyhalothrin metabolizing glutathione S-transferase CpGSTd1 from Cydia pomonella (L.). *Appl Microbiol Biotech* **98**, 8947-8962 (2014).

8 Tian, Z., Liu, J. & Zhang, Y. Structural insights into *Cydia pomonella* pheromone binding protein 2 mediated prediction of potentially active semiochemicals. *Sci Rep* **6**, doi:10.1038/srep22336 (2016).

9 Kilambi, K. P. & Gray, J. J. Rapid calculation of protein pK(a) values using Rosetta. *Biophys J* **103**, 587-595 (2012).
